# A combined subcision approach with either fractional CO_2_ laser (10,600 nm) or cross-linked hyaluronic acid versus subcision alone in atrophic post-acne scar treatment

**DOI:** 10.1007/s10103-022-03677-y

**Published:** 2022-12-24

**Authors:** Alaa Abdelaziz Abdelwahab, Ghada Abdel badea Omar, Mervat Hamdino

**Affiliations:** Dermatology and Venereology Department, Faculty of Medicine for Girls, Al-Azhar University, Cairo, Egypt

**Keywords:** Subcision, Fractional CO_2_ laser, Cross-linked HA filler, Atrophic acne scars

## Abstract

Different treatment options for post-acne scars exist, but with varying clinical efficacy, side effects, and prolonged downtime. This study aims to compare the efficacy and safety of combined subcision with either fractional CO_2_ laser or cross-linked hyaluronic acid filler (HA) versus subcision alone in the treatment of facial atrophic post-acne scars. Forty patients with atrophic post-acne scars were subjected to subcision on both sides of the face, then were randomly divided into three groups. Group I (20 patients): subcision combined with cross-linked HA filler injection at one side of the face; group II (20 patients): subcision followed by fractional CO_2_ at the other side of the face; and group III (20 patients): with subcision only as a control group. Treatment sessions were every month until clinical improvement or for maximum three sessions. The treatment’s efficacy was assessed by Goodman and Baron’s qualitative and quantitative grading systems. The two blinded investigator scores showed significant improvement in both the filler side versus subcision (*p* value = 0.015), and the fractional laser side versus subcision (*p* value < 0.001), with no statistically significant difference between both sides (*p* value = 0.171). Qualitative grading by Goodman and Baron scores showed that the percentage of patients with excellent improvement was higher in group 1 and group 2 than in group 3 with *p* value = 0.031; also the mean percentage of reduction in quantitative grading was higher in group 1 and group 2 than in group 3 with *p* value < 0.00. Either combined subcision with fractional CO_2_ laser or with cross-linked HA filler achieved superior improvement in facial atrophic post acne scars treatment with no serious side effects in this study. However, subcision only by blunt canula also had modest improvement.

## Introduction

Acne scarring has a significant detrimental impact on young adults’ quality of life [1]. Acne scars are divided into two categories: hypertrophic and atrophic. Icepick, rolling, and boxcar scars are the three types of atrophic acne scars [2].

There are several treatment options for atrophic acne scars, each with its own benefits and drawbacks. In order to improve the outcome, new procedures have been incorporated, and existing ones have been changed, as well as a combination of modalities is frequently required [3].

Subcision (subcutaneous incisionless surgery) is a surgical procedure that has been used for many years to treat depressed scars, wrinkles, and several types of atrophic acne scars, including rolling, superficial and deep boxcars, and pitted scars (except icepick scars) [4].

Fractional CO_2_ lasers can be used to treat moderate to severe atrophic acne scars with good variable results in macular, superficial, and medium depth scars, but deep scars and ice pick scars may improve only marginally. It can be used alone as monotherapy or combined with other procedures such as subcision [5].

Tissue injectables are materials injected into the depressed areas of the scar to elevate it to the level of the normal surrounding skin. Scars may be filled with hyaluronic acid (HA) (temporary filler), polylactic acid, or calcium hydroxyapatite (both are semi-permanent fillers) [6]. Consequently, we aimed to compare the efficacy and safety of combined subcision with either fractional CO_2_ laser or cross-linked HA filler versus subcision alone in the treatment of facial atrophic post-acne scars.

## Patients and methods

Forty patients with different types of atrophic post-acne scars were included in this study. All patients underwent subcision on both sides of the face, then patients were randomly divided into three groups: group I (20 patients)—subcision combined with cross-linked HA filler injection at one side of the face; group II (20 patients)—subcision followed by fractional CO_2_ at the other side of the face. Each side was randomly selected by choosing a sealed opaque envelope containing a card labeled with either laser or filler representing the right and left split face side treatments. Group III (20 patients): subcision only as a control group.

Patients were recruited from the Dermatology Outpatient Clinic in Al-Zahraa University Hospital, Cairo, Egypt, from August 2020 to December 2021*.* The study was done according to Declaration of Helsinki and approved by the Research Ethics Committee of Faculty of Medicine for Girls, Al-Azhar University, Cairo, Egypt, with approval code (202101579). All patients received complete information on the steps of treatment and written informed consent was obtained before participation in the study.

### Inclusion criteria

Inclusion criteria were adult patients of both sexes with mild to severe atrophic post-acne scars.

### Exclusion criteria

Patients less than 18 years old, pregnant and lactating women, patients with active inflammatory acne or active infection in the treatment area, patients with premalignant or malignant lesions in the treatment area, patients with photosensitive dermatoses, patients with a history of chronic diseases (coagulation defects and blood disease), patients with a history of keloid scars, patients using drugs causing photosensitivity or systemic retinoids in the previous 6 months, and patients who received HA filler in the previous year in the treatment area all were excluded*.*

Full history and dermatological examination was taken for detection of site, size, number, and type of acne scars.

Group I (filler side): patients underwent one session of subcision, followed by cross-linked HA filler injection on one side of the face.

Group II (fractional side): patients underwent one session of subcision, followed by fractional CO_2_ laser on the other side of the face every month for a maximum of three sessions or until clinical improvement has been achieved.

Group III (Subcision only): patients were subjected to one session of subcision alone.

Each patient’s entire face was disinfected with alcohol and cleaned with saline. Anesthesia was injected subcutaneously with 2% lidocaine, then a sharp needle was inserted at a shallow angle, with the blade facing upward, at the periphery of the scarred area to create a pathway to facilitate the entry of the blunt cannula.

Subcision was performed once with an 18-gauge blunt cannula introduced at a 30° angle into the mid to deep dermis, then moved back and forth in a fan-like motion under the scar to release fibrous bands at the dermal or deep dermal, subcutaneous plane until the sound of bands breaking was lost, and no resistance was felt (endpoint). To establish hemostasis, firm pressure was applied for at least 5 min. After subcision, the face was cleaned with alcohol swabs and then sterile saline, and the facial halves were randomized to receive treatments in groups I and II.

Filler side (group I): one side of the face was injected with cross-linked HA filler of mild viscosity (STYLAGE XL 26 mg/ml; VIVACY France), into each scar using 27G needle gauge introduced both in a parallel plane for broader lesions and in a perpendicular plane into the superficial to mid dermis with total injected dose ranged from 0.5 to 1 ml of HA according to scars number, depth, and width.

Fractional side (group II): the other side of the face was treated with a fractional CO_2_ laser (10,600 nm) using (Smart Xide DOT device, Deka, Florence, Italy) with the following parameters: 15-W power, spacing of 800 μm, dwell time 600 μs, and stack level (2–3) in each session with fine adjustment according to skin type and patient’s reactions. In order to ensure minimum pain during the laser session, topical anesthesia (Pridocaine cream®, lidocaine 2.5%, and prilocaine 2.5%) was applied 30 min before treatment.

All patients were instructed to avoid sunlight exposure and to use sunscreen (SPF ≥ 50).

### Assessment

Photographic evaluations were performed on several profiles before each session, 1 month, and 6 months after the last session, all with identical camera settings (Huawei P20 Pro 40 megapixels digital camera, model CLT-L29, China).

Evaluation of global response to treatment by two blinded investigators, using patient's photographs before and after treatment by a quartile grading scale (0 = slight improvement, < 25, 1 = moderate improvement 25–49%, 2 = significant improvement 50–74%, and 3 marked improvement > 75%).

The physician’s clinical assessment was performed for all groups by counting and grading scars at the baseline, 1 month after the last session, and 6 months after the last session. Any changes in scar grading were documented using the Goodman and Baron qualitative acne scarring grading system [7]. If the grade was reduced by 2, it was rated excellent; if it was reduced by 1, it was considered good; and if there was no grade reduction, it was rated poor [8]. A quantitative acne scarring grading system developed by Goodman and Baron [9] was used to measure progress. If the scale was lowered by 0–5 points, the reduction was considered minimal. If the reduction was 5–10 points, it was considered moderate reduction; if the reduction was 10–15 points, it was considered good reduction; and if the scale was reduced by more than 15 points, it was considered very good reduction [10].

Subjective evaluation of the patient feedback was performed at the end of the study to rate the improvement of each side of the face using the quartile scale (0 = slight improvement < 25%, 1 = moderate improvement 25–49%, 2 = significant improvement 50–74%, and 3 = marked improvement > 75%) [11]. In addition, the physician asked them verbally and examined side effects such as downtime, erythema, edema, post-inflammatory hyperpigmentation, pain, and others like milia, infections, and pain scoring using the numeric pain rating scale [12].

### Statistical analysis

Data were analyzed through the Statistical Package for Social Science (IBM SPSS, Armonk, NY, IBM Corp.) version 23. The quantitative data were presented as mean, standard deviations, and ranges when their distribution was found parametric and median with inter-quartile range (IQR) when non-parametric. Additionally, qualitative variables were presented as numbers and percentages. *p* value was considered significant when *P* < 0.05.

## Results

This study included 40 patients (19 females and 21 males) with different types of atrophic facial post acne scars, aged from 18 to 40 years. Demographic data of participants are demonstrated in Table 1.

**Table 1:** Demographic data and characteristics of the studied patients

Variable		(*n* = 40)
Age (years)	Mean ± SD	27.93 ± 6.05
	Range	18–40
Sex	Female	19 (47.5%)
	Male	21 (52.5%)
Scar type	Mixed	19 (31.7%)
	Rolling + icepick	19 (31.7%)
	Rolling + boxcar	13 (21.7%)
	Rolling	3 (5.0%)
	Boxcar	2 (3.3%)
	Boxcar + icepick	3 (5.0%)
	Icepick	1 (1.7%)
Scar duration (years)	Mean ± SD	5.5 (3.5–7)
	Range	2–17
Skin type	II	11 (27.5%)
	III	16 (40.0%)
	IV	13 (32.5%)
Family history	Negative	16 (40.0%)
	Positive	24 (60.0%)

*SD* standard deviation

The two blinded investigator scores 6 months after treatment showed significant improvement in the filler side versus subcision (*p* value = 0.015), highly statistically significant improvement in the fractional laser side versus subcision (*p* value < 0.001), while no statistically significant difference was detected between the HA filler side and fractional CO_2_ laser side (*p* value = 0.171) (Table 2).

**Table 2:** Comparison between the three studied groups regarding blind investigator score

Blind investigator	Group 1 (filler side)	Group 2 (fractional laser side)	Group 3 (subcision)	Test value	*p* value
	No. = 20	No. = 20	No. = 20		
Mean ± SD	2.35 ± 0.75	2.65 ± 0.59	1.6 ± 0.99	13.543^≠^	0.001
Median (IQR)	2.5 (2–3)	3 (2–3)	2 (1–2)		
Range	1–3	1–3	0–3		
Slight improvement (< 25%)	0 (0.0%)	0 (0.0%)	3 (15.0%)	15.65*	0.016
Moderate improvement (25–49%)	3 (15.0%)	1 (5.0%)	6 (30.0%)		
Significant improvement (50–74%)	7 (35.0%)	5 (25.0%)	7 (35.0%)		
Marked improvement (> 75%)	10 (50.0%)	14 (70.0%)	4 (20.0%)		
Post hoc analysis					
Filler vs fractional laser	Group 1 vs group 3		Group 2 vs group 3		
0.171	0.015		< 0.001		

*p* value > 0.05: non-significant; *p* value < 0.05: significant; *p* value < 0.01: highly significant

^*^Chi-square test; ^≠^Kruskal–Wallis test

Qualitative Goodman and Baron (Severity assessment) demonstrated a statistically significant difference between the three studied groups after 6 months of treatment with *p* value < 0.001. Also, the improvement of qualitative grading after 6 months showed that the percentage of patients with excellent improvement was higher in group 1 and group 2 than in group 3 with *p* value = 0.031 (Table 3).

**Table 3:** Comparison between the three studied groups regarding qualitative grading before and 6 months after treatment and the improvement of qualitative grading 6 months after treatment

Qualitative grading		Group 1 (filler side)	Group 2 (fractional laser side)	Group 3 (subcision)	Test value	*p* value
Before	Grade I	0 (0.0%)	0 (0.0%)	0 (0.0%)	0.857*	0.931
	Grade II	2 (10.0%)	1 (5.0%)	1 (5.0%)		
	Grade III	10 (50.0%)	9 (45.0%)	9 (45.0%)		
	Grade IV	8 (40.0%)	10 (50.0%)	10 (50.0%)		
After 6 m	Grade I	0 (0.0%)	4 (20.0%)	1 (5.0%)	25.050*	0.000
	Grade II	18 (90.0%)	15 (75.0%)	7 (35.0%)		
	Grade III	2 (10.0%)	1 (5.0%)	11 (55.0%)		
	Grade IV	0 (0.0%)	0 (0.0%)	1 (5.0%)		
Qualitative grading Improvement after 6 months	Poor	3 (15.0%)	1 (5.0%)	8 (40.0%)	10.671*	0.031
	Good	11 (55.0%)	9 (45.0%)	9 (45.0%)		
	Excellent	6 (30.0%)	10 (50.0%)	3 (15.0%)		
Chi-square test	***X***^2^	26.133	32.650	13.064		
	***p*** value	** <** 0.001	** < **0.001	0.005		

*p* value > 0.05: non-significant; *p* value < 0.05: significant; *p* value < 0.01: highly significant

^*^Chi-square test

Quantitative Goodman and Baron scores showed statistically significant difference between the three studied groups 6 months after treatment with *p* value < 0.001. The mean percentage of reduction in quantitative grading was higher in group 1 and group 2 (62.29 ± 13.62 and 68.58 ± 10.04; respectively) than in group 3 (44.29 ± 16.45) with *p* value < 0.001 and the grading of reduction also was higher in group 1 and 2 than in group 3 with *p* value = 0.004. Comparing the post-treatment results, the fractional CO_2_ laser side showed a better clinical outcome than the HA filler side (Table 4).

**Table 4:** Comparison between the three studied groups regarding quantitative grading before and 6 months after treatment and the improvement of quantitative grading 6 months after treatment

Quantitative grading		Group 1 (Filler side)	Group 2 (Fractional laser side)	Group 3 (Subcision)	Test value	*p* value
		No. = 20	No. = 20	No. = 20		
Before	Mean ± SD	17.65 ± 5.49	18.7 ± 5.2	16.8 ± 4.56	1.846 ≠	0.397
	Median (IQR)	17 (14–20.5)	18 (15–21)	16 (14–19)		
	Range	10–34	12–34	12–30		
6 months after	Mean ± SD	6.55 ± 3.12	5.75 ± 2.24	9 ± 2.53	16.980 ≠	0.000
	Median (IQR)	6 (5–7.5)	5 (4–6)	8 (8–10)		
	Range	3–17	3–11	5–16		
Quantitative grading Reduction After 6 months	Mean ± SD	62.29 ± 13.62	68.58 ± 10.04	44.29 ± 16.45	17.115•	0.000
	Range	32–85	50–88	15.79–75		
	Minimal	1 (5.0%)	0 (0.0%)	6 (30.0%)	18.857*	0.004
	Moderate	8 (40.0%)	5 (25.0%)	11 (55.0%)		
	Good	8 (40.0%)	11 (55.0%)	2 (10.0%)		
	Very good	3 (15.0%)	4 (20.0%)	1 (5.0%)		
Wilcoxon rank test	Test value	** − **3.927	** − **3.927	** − **3.925		
	***p*** value	** < **0.001	** < **0.001	** < **0.001		
Post hoc analysis						
	Group 1 vs group 2	Group 1 vs group 3		Group 2 vs group 3		
6 months after	0.314	0.001		** < **0.001		
% of reduction after 6 m	0.150	** < **0.001		** < **0.001		

*p* value > 0.05: non-significant; *p* value < 0.05: significant; *p* value < 0.01: highly significant

^*^Chi-square test; •one-way ANOVA test; ^≠^Kruskal–Wallis test

**Fig. 1:**
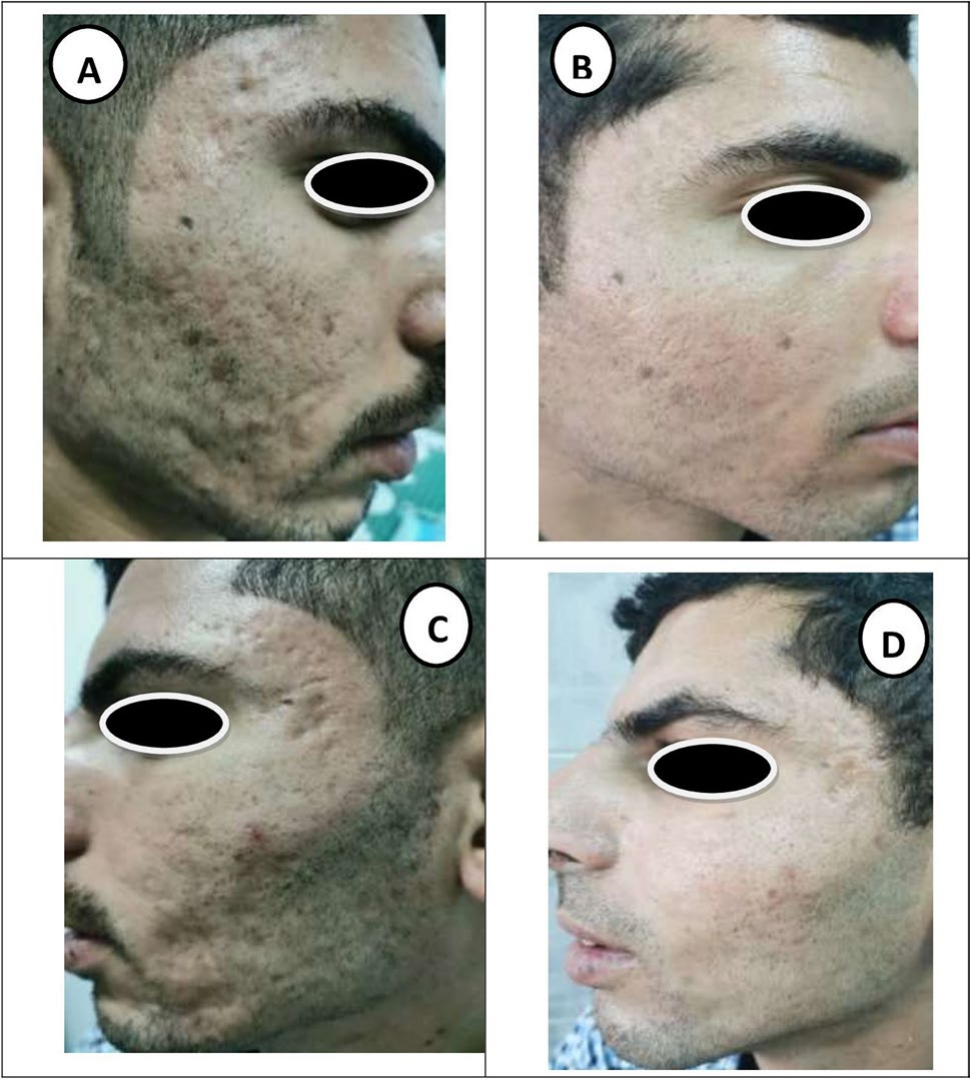
A 27-year-old male patient with mixed type atrophic acne scars: **A** laser side before treatment (grade IV). **B** Laser side 6 months after treatment (grade II). **C** HA filler side before treatment (grade IV). **D** HA filler side 6 months after treatment (grade II)

**Fig. 2:**
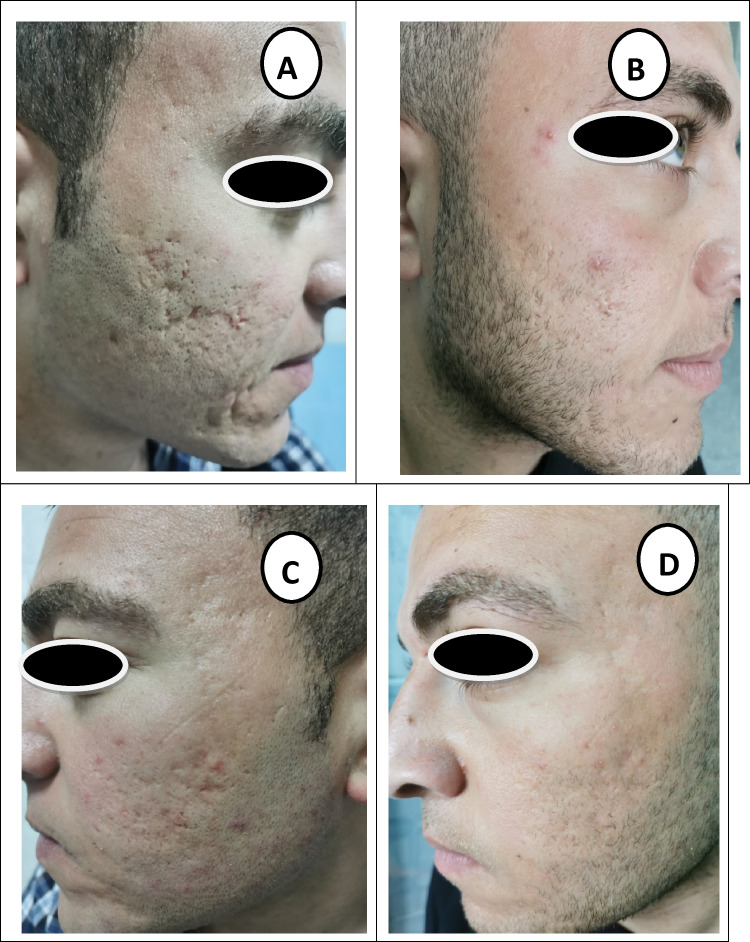
A 21-year-old male patient with mixed type atrophic acne scars: **A** laser side before treatment (grade IV). **B** Laser side 6 months after treatment (grade II). **C** HA filler side before treatment (grade III). **D** HA filler side 6 months after treatment (grade II)

**Fig. 3:**
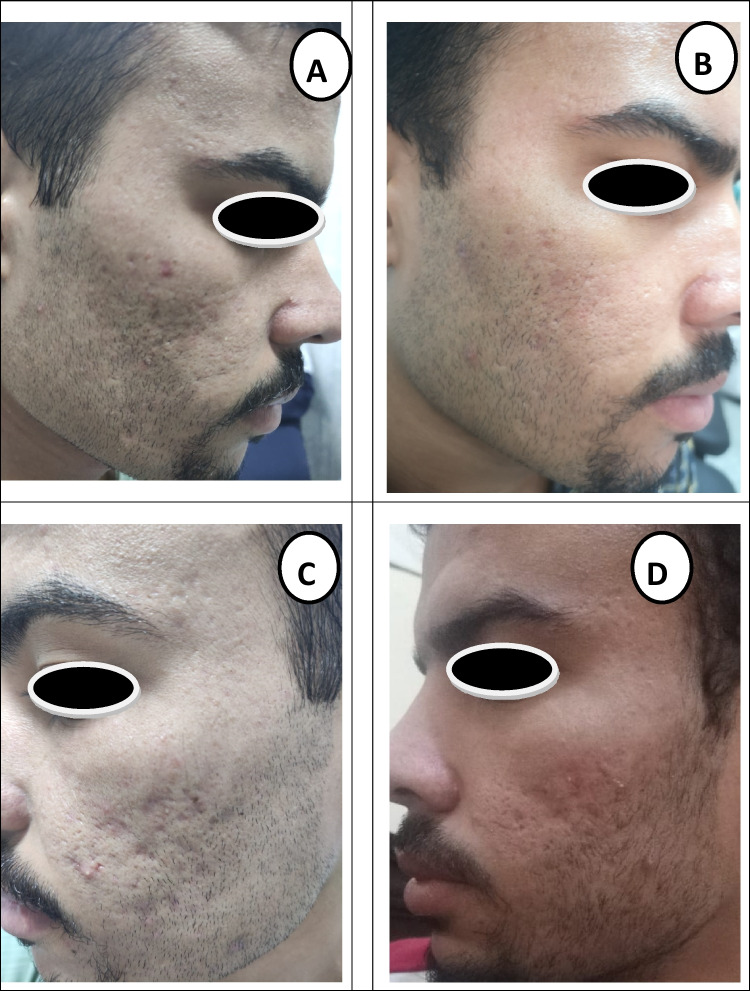
A 26-year-old male patient with mixed type atrophic acne scars treated with subcision only: **A** before treatment (grade III). **B** 6 months after treatment (grade II). **C** Before treatment (grade III). **D** 6 months after treatment (grade II)

Clinical photos of patients at baseline and after 6 months of follow-up showing marked improvement of mixed types of atrophic post-acne scars in all groups (Figs. 1, 2 and 3).

The present results showed a higher patient satisfaction in group 1 and 2 than in group 3 (*p* value 0.011, < 0.001 respectively) (Table 5).

**Table 5:** Comparison between the three studied groups regarding patient satisfaction

Pt. satisfaction	Group 1 (filler side)		Group 2 (fractional laser side)	Group 3 (subcision)		Test value	*p* value
	No. = 20		No. = 20	No. = 20			
Mean ± SD	2.25 ± 0.72		2.6 ± 0.6	1.5 ± 0.95		15.187^≠^	0.001
Median (IQR)	2 (2–3)		3 (2–3)	1.5 (1–2)			
Range	1–3		1–3	0–3			
Slight improvement (< 25%)	0 (0.0%)		0 (0.0%)	3 (15.0%)		17.977*	0.006
Moderate improvement (25–49%)	3 (15.0%)		1 (5.0%)	7 (35.0%)			
Significant improvement (50–74%)	9 (45.0%)		6 (30.0%)	7 (35.0%)			
Marked improvement (> 75%)	8 (40.0%)		13 (65.0%)	3 (15.0%)			
Post hoc analysis							
Group 1 vs group 2		Group 1 vs group 3			Group 2 vs group 3		
0.100 (NS)		0.011 (S)			< 0.001 (HS)		

*p* value > 0.05: non-significant; *p* value < 0.05: significant; *p* value < 0.01: highly significant

^*^Chi-square test; ^≠^Kruskal–Wallis test

**Fig. 4:**
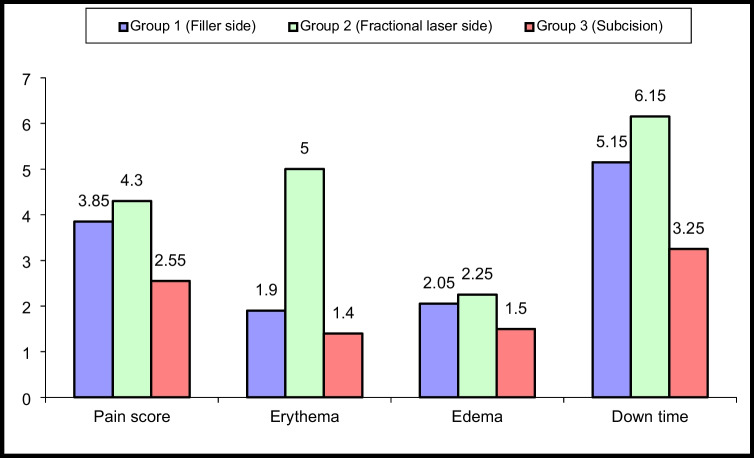
Comparison between the three studied groups regarding side effects

A statistically significant difference was found between the studied groups in terms of pain, erythema, edema, and downtime found lower in the subcision group than filler and fractional laser treated sides with *p* value = 0.005 for pain score, *p* value < 0.001 for erythema, *p* value = 0.005 for edema, and downtime with a *p* value < 0.001 (Fig. 4).

## Discussion

Treatment of acne scarring continuously represents an innovative area for research. A combination of modalities is frequently necessary to get the most significant outcomes [3]. Our results showed a statistically significant improvement in both the fractional laser side, and the filler side versus subcision alone while no statistically significant difference was detected between the HA filler side and fractional CO_2_ laser side (*p* value = 0.171) by the two blinded investigator score 6 months after treatment.

Results of this study also demonstrated that the mean percentage of reduction in quantitative grading was higher in group 1 and group 2 than in group 3 with *p* value < 0.001 and the grading of reduction also was higher in group 1 and 2 than in group 3 with *p* value = 0.004.

Also, the improvement of qualitative grading after 6 months showed that the percentage of patients with excellent improvement was higher in group 1 and group 2 than in group 3 with *p* value = 0.031. Fractional CO_2_ laser-treated side showed a better clinical outcome than the HA filler side as 50.0% showed excellent improvement (two grade reduction) compared with 30.0% in the HA filler side, whereas the subcision group showed 15.0% with excellent improvement.

These findings are similar to Nilforoushzadeh et al. [13], who conducted a comparative study on 30 patients who received five sessions of fractional CO_2_ laser alone on one side of the face versus combined fractional CO_2_ laser and subcision on the other side. After 6 months of therapy, the combined approach revealed a higher recovery rate (54.7%) and a more patient satisfaction.

Despite applying fewer laser sessions, our study showed a higher percentage of improvement than the study of Nilforoushzadeh et al. [13], which can be attributed to the blunt cannula used in our work compared to an insulin needle used for subcision in their work. In addition, Nilforoushzadeh et al. study showed bruising and post-inflammatory hyperpigmentation in their combination method but not in ours, demonstrating the superior healing effect of subcision done by the blunt cannula.

In addition, our findings agreed with Gheisari et al. [14], who reported that subcision using a blunt cannula is more effective and has fewer side effects than the Nokor needle for acne scars treatment.

In addition, Kareem et al. [15] compared fractional carbon dioxide laser resurfacing alone versus combined with subcision by CO_2_ gas and demonstrated more improvement with the combined method, with 10% showing good improvement compared to (50.0%) with excellent improvement in our study. A higher percentage of excellent improvement in our study compared with their study may be due to subcision using a blunt cannula being more effective than subcision by CO_2_ gas.

Another study conducted by Al‐Dhalimi and Arnoos [16] revealed adverse effects such as edemas, hematomas, pain, post-inflammatory hyperpigmentation, and fibrotic nodules observed immediately after the procedure of subcision. In our study, the side effects detected after treatment were edema, pain, and erythema, which lasted for 3–10 days in most patients.

Few studies were about the use of dermal fillers in treating acne scars, although there is evidence supporting the use of poly‐l‐lactic acid and HA fillers to treat acne scars [17]. Using HA fillers in atrophic acne scars has gained interest from the patients and physicians. HA fillers are differing in their physical properties and time of tissue residence [18, 19].

Balighi et al. [20] carried out a split-face study on 20 patients. They all underwent subcision with an 18‐gauge needle on one side of the face and subcision in conjunction with insertion of a subdermal implant on the other side of the face. They revealed mild improvement in about 60% of cases with subcision, but there was no significant difference with the use of a subdermal implant. However, a higher percentage of improvement in our study may be due to the different types of subdermal implants used, as fillers vary substantially in terms of cross-linking method, percentage of cross-linking, viscosity, hardness, HA concentration, amount of HA, and bound water, gel-to-fluid ratio, degree of HA modification, particle size, ease of injection, and indications. All of these variables contribute to the treatment’s flexibility with fillers.

Hasegawa et al. [21] did a study involving the treatment of acne scarring using fractional carbon dioxide laser as a single treatment modality. It showed excellent, good, fair, and poor responses in 6.5%, 29%, 35.5%, and 29% of patients, respectively. In a similar study conducted by Zhang et al. [22] using fractional carbon dioxide laser, 66.4%, 30%, 3.7%, and 0.9% of patients reported grades 1, 2, 3, and 4 improvements.

Goodman and Van Den Broek [23] used Hyaluronic acid fillers as a single therapy method on five patients with atrophic acne scarring. After two sessions, the mean scar count dropped from 48.8 at baseline to 15.4. Moreover, 1 month following the second therapy, the mean grade dropped from 3.2 to 2.6, indicating improvement.

In their study on patients with acne scars, Nilforoushzadeh et al. [24] performed the subcision technique on a total of eight patients, utilizing cannulae numbers 18 and 21 for two sessions, with an objective assessment of the treatment effect performed by a blinded dermatologist. Approximately 88% of patients improved and were pleased with the therapy outcomes.

The degree of scar improvement following subcision appears to be time-dependent, with greater improvement occurring as time passes. Scar remodeling is a continual process that does not reach a stable state until at least 2 years after subcision [25]. Our patients demonstrated significant improvement 2 days after subcision; however, this was temporary due to edema. Although the progress slowed when the edema went away, it returned 1 month later, owing to collagen remodeling.

Since the skin following laser procedures may be more vulnerable to ultraviolet sources due to lower levels of keratinization and reduced melanin content, as well as the lack of postoperative maneuvers by the patients, there was a brief period of erythema that demonstrated a significant difference between the laser-treated side and the HA-treated side in our study. Hyaluronic acid, on the contrary, has a pro-angiogenic and anti-angiogenic equilibrium [26].

Edema was more noticeable in the HA side than the laser side since HA is a highly hygroscopic (moisture-retaining) molecule, which results in a hydrated matrix [27].

## Conclusion

Both combination modalities demonstrated a significant improvement in all scar types. The fractional CO_2_ laser–treated side showed better clinical outcomes than the HA Filler side, but the difference between both sides was statistically non-significant.

### Limitations

The small number of participants calls for more controlled studies evaluating these therapeutic options to validate its efficacy in a larger cohort of patients.

## Data Availability

The data that support the findings of this study are openly available in Google scholar and PubMed at http://doi.org/[doi], reference number 28.
